# Assessment of initiation of breastfeeding practice in Kassala, Eastern Sudan: a community-based study

**DOI:** 10.1186/s13006-018-0177-6

**Published:** 2018-07-25

**Authors:** Ahmed A. Hassan, Zainab Taha, Mohammed Ahmed A. Ahmed, Abdel Aziem A. Ali, Ishag Adam

**Affiliations:** 1 Faculty of Medicine, University of Khartoum, Khartoum, Sudan; 2 College of Natural and Health Sciences, Zayed University, Dubai, United Arab Emirates; 3 Faculty of Medicine, Gadarif University, Al Qadarif, Sudan; 4 Faculty of Medicine, Kassala University, Kassala, Sudan

**Keywords:** Timely initiation of breastfeeding, Early initiation of breastfeeding, Maternal medical disorders, Sudan

## Abstract

**Background:**

The World Health Organization (WHO) encourages early initiation of breastfeeding within the first hour after birth with the objective of saving children’s lives. There are few published research papers about factors associated with the initiation of breastfeeding in Sudan.

The aim of this study was to investigate the prevalence of and factors associated with the timely initiation of breastfeeding among mothers with children two years and under in Kassala, Eastern Sudan.

**Methods:**

A community-based cross-sectional study was conducted from December 2016 to March 2017. Mothers were interviewed using a structured questionnaire.

**Results:**

A total of 250 mother-child pairs participated in the study. The mean (standard deviation) of maternal age and children’s age was 27.1 (5.68) years and 11.9 (6.9) months, respectively.

Of the 250 mothers, 218 (87.2%) initiated breastfeeding within the first hour. In multivariable logistic regression analysis, factors associated with the delay of breastfeeding initiation were having a male baby (Adjusted Odds Ratio [AOR] 3.90, 95% Confidence Interval [CI]1.33, 11.47), and mothers with medical disorders (AOR 5.07, 95% CI 1.22, 21.16).

**Conclusion:**

There was a high prevalence of early initiation of breastfeeding. An association with delayed initiation of breastfeeding was found amongst mothers who had medical disorders and those who had a male infant. Wherever possible, early initiation of breastfeeding should be promoted for all infants, regardless of gender.

## Background

According to the United Nations Children’s’ Fund (UNICEF) [1], the first 1000 days of a child’s life (9 months of pregnancy plus the first 2 years of life) is considered to be a crucial period. In countries with fewer resources such as Sudan, children two years and under are the most vulnerable in terms of morbidity and mortality [2, 3]. These children are vulnerable to malnutrition, gastroenteritis and respiratory tract infections [2–6]. A study published in 2015 documented high rates of malnutrition among children in Khartoum who were either not breastfed or were weaned early [6]. The children of Kassala are categorized as among the most vulnerable in Sudan where high rates of acute and chronic malnutrition were reported, especially among children [7]. The main reported causes of childhood hospitalisation in previous studies which include Sudan were respiratory tract infections and gastroenteritis [2, 8–12]. Predictors of hospital admission were identified as non-exclusive breastfeeding, delayed initiation of breastfeeding, maternal unemployment, having two or more children, complementary feeds given by a person other than the mother, prolonged rupture of membranes, home delivery, intrapartum fever, and Apgar score < 7 at 5 min [2, 8–13]. Likewise, early initiation of breastfeeding and exclusive breastfeeding are documented as key predictors of infant survival [14, 15].

Aiming to save children’s lives, the World Health Organization (WHO) developed a set of recommendations, including: Skin-to-skin’ at or as soon as possible after the birth, and unrestricted access to the breast thereafter to enhance the initiation of breastfeeding and the establishment of an adequate milk supply [16]. The WHO rated initiation of breastfeeding based on the percentage of babies breastfed within one hour of birth as follows: (0–29%), (30–49%), (50–89%), (90–100%) to poor, fair, good and very good, respectively [17]. Varying rates of initiation of breastfeeding were 17.7 and 98.4% [18–22].

Early initiation of breastfeeding promotes exclusive breastfeeding by enhancing bonding, increasing the likelihood of breastfeeding success, and generally extending breastfeeding duration [16, 17, 23].

Poor breastfeeding practices such as delayed initiation, low rates of exclusivity, and early weaning have been documented in previous studies undertaken in different regions of Sudan including Kassala [5, 24–26]. In addition to more recent reports these practices have been noted previously. A 1993 publication identified one urban and rural community study including the eastern region of Sudan where 27.1% of mothers weaned their infants before 12 months [27].

Although many studies in Sudan identify delayed breastfeeding initiation [19, 28], none report the factors associated with initiation of breastfeeding. Moreover, most of the available data about breastfeeding in Sudan is hospital-based [4, 27, 28] Investigating initiation of breastfeeding at community-level would help identify the factors which lead to early weaning and delayed breastfeeding initiation. Community based research is more efficient in yielding data needed for future community-based interventions.

The aim of this study is to investigate the prevalence of breastfeeding initiation and associated factors among mothers of infants aged two and under in Kassala, Eastern Sudan.

## Methods

A two-stage random cluster study was conducted in Kassala, Eastern Sudan between December 2016 and March 2017. Stage one, simple random sampling of the localities was performed to randomly identify households. Similarly, stage two, random sampling of the household was used to identify participants (children aged two years or less). Kassala has an estimated population of 453,159 inhabitants of which 55% live in urban areas, with 33,604 and 52,853 households in urban and rural areas, respectively [29]. Houses were mapped to select a representative sample. The main tool used to collect data in this study was a structured questionnaire. Three female medical officers were trained by the investigators to collect the data. Before data collection the questionnaire was tested among 10 mothers (not included in the final sample) and the necessary corrections were done. The following inclusion criteria were set before conducting the study: willingness to participate, having a child of two years or younger (where there were two children aged two or younger, the interview was based on the youngest child), and availability at the time of data collection.

The time for the initiation of breastfeeding was measured by asking the mother about the time at which her current child was put to the breast after delivery (within one hour or more than one hour of delivery) [16, 19]. Initiation of breastfeeding with one hour or more than one hour was considered as early and delayed initiation respectively. The questionnaire was coded with the outcome variable (initiation of breastfeeding) as (0) and (1) for early and delayed initiation of breastfeeding, respectively.

Eligible participants were approached and the purpose of the study explained. Participants signed the study consent form after assurance was provided regarding the confidentiality of any information gained and the right to withdraw at any time clarified. After the participant’s acceptance and fulfilment of the study inclusion criteria, a face-to-face interview was conducted using a structured questionnaire.

The questionnaire included demographic information (maternal age, education, occupation), place of delivery (home, institutional) mode of delivery (vaginal delivery, caesarean delivery), and medical disorders such as diabetes in pregnancy, hypertension in pregnancy and asthma, delivery details; infant information (e.g. age in months, gender, child order, history of hospitalization, and the main reason for hospitalization.

Breastfeeding information about the index child was collected (e.g. initiation of breastfeeding, breastfeeding difficulty, etc.).

The calculated sample size was 250 participants using an assumption of 79.6% prevalence of early initiation of breastfeeding [30]. This sample was selected to give 80% power with a precision of 5%.

### Statistical analysis

After the data were collected, the questionnaires were checked and all participants (*n* = 250) found to have complete data. Data were entered into the computer using Statistical Package for the Social Science (SPSS0 version 20.0 for Windows) and double checked before analysis. The results were illustrated in tables and text calculating the means and standard deviation (SD) for continuous variables, frequencies and percentages for categorical variables to describe participant response. T-test and Chi-square test were applied to analyse continuous and categorical data, respectively. Variables with *P*-value of < 0.2 in univariate analysis were entered in multivariable logistic analysis with delayed initiation of breastfeeding as the dependent variable and the other variables such as age, birth order, education, residence, maternal medical disorders, mode of delivery, child gender, etc. as the independent variables. Odds Ratio (OR) and 95% Confidence Intervals (CI) were calculated. *P*-value < 0.05 was considered as significant.

## Results

A total of 250 mother-child pairs participated in the study. The mean (SD) of maternal and infant age was 27.1 (5.68) years and 11.9 (6.9) months, respectively. Maternal age ranged from 13 to 40 years and 40 (16%) of the women were ≤ 20 years. Twenty seven percent of participants were primiparous.

Of the 250 participants, 218 (87.2%) initiated breastfeeding within the first hour of delivery. Of 218 mothers who initiated breastfeeding within 1 h, 165 (66%) did so immediately and the remainder 53 (21.2%) within 1 h.

Only 11.2% (28/250) of the mothers were employed (government employed (*n* = 26), private (*n* = 1), own business (*n* = 1)). Almost all (*n* = 27) mentioned that there was no lactation room in their work place and 6/28 (21.4%) of the employed mothers complained about the lack of cooperation of their employers regarding breastfeeding issues (providing flexible work hours). One-fifth of the participants, 55 (22%), faced breastfeeding problems such as nipple problems, and mastitis.

Of the 250 participants, 103 (41.2%) were rural residents, 193 (77.2%) were housewives and 171 (68.4%) had received less than secondary level education. Twenty–three (9.2%) of the mothers had medical disorders (diabetes in pregnancy (*n* = 2), hypertension in pregnancy including pre-eclampsia (*n* = 5) and other (*n* = 16). Less than half 114 (45.6%) of the children were females. Over three-quarters of the children, 207 (82.8%) were delivered vaginally and the remainder 43 (17.2%) by caesarean section. Ninety four percent of infants born vaginally initiated breastfeeding within the first hour of delivery compared to 77% of infants born by caesarean section (*p* = 0.024).

One-hundred and forty six (58.4%) of the mothers had received breastfeeding education provided by healthcare personnel during pregnancy and/or after the delivery.

About one-third, (88/250) 35.2%, of the children were hospitalized at least once in the last 6 months and spent 24 h or more at the hospital. The most common reasons for hospitalization were respiratory tract infections, gastroenteritis, and malaria. Ninety three percent of infants hospitalized had initiated breastfeeding within the first hour of delivery, compared to 84% who weren’t hospitalized (*p* = 0.052).

The child age, number of children less than five years, residence, mode of delivery, having received breastfeeding education, and paternal education level and occupation were found to be associated with initiation of breastfeeding in bivariate analysis only (Table 1).

**Table 1:** Characteristics of participants in Kassala, Eastern Sudan

Variables		Total		Initiation of breastfeeding Early (*n* = 218) Delayed (*n* = 32)					
		Mean (SD)		Mean (SD)		Mean (SD)		Odds Ratio (95% Confidence Interval)	*P*-value
Maternal age, years		27.1 (5.7)		26.9(5.65)		28.3 (5.8)		1.04(0.97, 1.11)	0.186
Child age, months		11.9 (6.9)		12.2 (6.8)		9.2 (6.86)		0.93(0.88, 0.99)	0.019
Birth order		2.4(1.5)		2.4(1.5)		2.0(1.46)		0.81(0.60, 1.09)	0.179
Number of children < 5 years		1.7(0.7)		1.7(0.73)		1.4(0.66)		0.45(0.24, 0.85)	0.012
Number of breastfeeding per day		7.4(3.4)		7.5(3.58)		6.2(1.68)		0.87(0.76, 0.99)	0.442
		N	%	N	%	N	%	OR (95% CI)	*P*-value
Child gender	Male	136	54.4	111	81.6	25	18.4	0.29 (0.12, 0.70)	0.004
	Female	114	45.6	107	93.9	7	6.1		
Residence	Rural	103	41.2	95	92.2	8	7.8	2.29 (0.98, 5.33)	0.054
	Urban	147	58.8	123	83.7	24	16.3		
Living with extended family	Yes	126	50.4	109	86.5	17	13.5	0.88 (0.42, 1.85)	0.741
	No	124	49.6	109	87.9	15	12.1		
Mode of delivery	Vaginal	207	82.8	185	89.4	22	10.6	2.54 (1.10,5.86)	0.024
	Caesarean	43	17.2	33	76.7	10	23.3		
Place of delivery	Institutional	136	54.4	114	83.8	22	16.2	0.49 (0.22, 1.10)	0.081
	Home	114	45.6	104	91.2	10	8.8		
Received education on breastfeeding by healthcare personnel	Yes	146	58.4	120	82.2	26	17.8	0.29 (0.11, 0.73)	0.006
	No	104	41.6	98	94.2	6	5.8		
Faced breastfeeding difficulties	Yes	55	22	50	90.9	5	9.1	1.44 (0.52, 3.96)	0.472
	No	195	78	168	86.2	27	13.8		
Maternal education	<Secondary level	171	68.4	149	87.1	22	12.9	0.98 (0.44, 2.18)	0.964
	≥Secondary level	79	31.6	69	87.3	10	12.7		
Paternal education	<Secondary level	145	58.0	133	91.7	12	8.3	2.60 (1.21, 5.60)	0.012
	≥Secondary level	105	42.0	85	81.0	20	19.0		
Maternal medical disorders	Yes	23	9.2	16	69.6	7	30.4	0.28 (0.10, 0.75)	0.008
	No	227	90.8	202	89.0	25	11.0		
Maternal occupation	Housewives	193	77.2	165	85.5	28	14.5	0.44 (0.14, 1.32)	0.137
	Employed	57	22.8	53	93.0	4	7.0		
Paternal occupation	Employed	124	49.6	116	93.6	8	6.5	3.41 (1.46, 7.92)	0.003
	Non-employed	126	50.4	102	81.0	24	19.0		
Child hospitalization	Yes	88	35.2	82	93.2	6	6.8	2.45 (0.96, 6.22)	0.052
	No	162	64.8	136	84.0	26	16.0		

In multivariable logistic regression analysis (Table 2), factors associated with the delay of breastfeeding initiation were male baby (Adjusted Odds Ratio [AOR] 3.90, 95% CI 1.33, 11.47), and maternal medical disorders (AOR 5.07, 95% CI 1.22, 21.16).

**Table 2:** Multivariable logistic regression analyses of factors associated with delay initiation of breastfeeding among mothers with child of two years and less in Kassala, Eastern Sudan

Variables		Adjusted Odds Ratio	95% Confidence Interval	*P*-value
Maternal age, years		1.07	0.97, 1.19	0.185
Child age, months		0.95	0.88, 1.02	0.122
Birth order		0.75	0.49, 1.15	0.181
Number of children < 5 years		0.61	0.29, 1.27	0.187
Child gender	Male	3.90	1.33, 11.47	0.013
	Female	Reference		
Residence	Rural	0.75	0.24, 2.28	0.606
	Urban	Reference		
Mode of delivery	Vaginal delivery	0.63	0.17, 2.30	0.483
	Caesarean delivery	Reference		
Place of delivery	Institutional	2.36	0.78, 7.19	0.130
	Home	Reference		
Received education on breastfeeding by healthcare personnel	Yes	1.42	0.41, 4.92	0.583
	No	Reference		
Paternal education	< Secondary level	0.48	0.16, 1.44	0.192
	≥ Secondary level	Reference		
Maternal medical disorders	With	5.07	1.22, 21.16	0.026
	Without	Reference		
Maternal occupation	Housewives	1.58	0.39, 6.44	0.527
	Employed	Reference		
Paternal occupation	Employed	0.34	0.11, 1.05	0.061
	Not employed	Reference		
Child hospitalization	Yes	1.47	0.45, 4.84	0.523
	No	Reference		

## Discussion

The main finding of the current study was the rate of early initiation of breastfeeding (87.2%) within one hour of delivery. This rate was higher compared with the national rate (68.7%) in Sudan [19], Eastern Sudan (45.8–76.6%) [28, 31]. This rate (87.2%) is recognized by the WHO as “good” (between 50 and 89%) [17], but is low compared with 92.8% in Khartoum [4] and 90.6% in Kassala, in eastern Sudan [26]. Variations in breastfeeding rates even within the same country have been reported before in Ethiopia [32], where the highest rate was reported in Addis Ababa, (71.5%), and the lowest, (41.7%). in the Somali regional state. Low rates of early initiation of breastfeeding have been reported in other African countries, 34.7–78.3% in Nigeria [20, 21] and 51.8% in Uganda [22].

The current study showed that male infants were 3.9 times more likely to experience delayed breastfeeding initiation. This is in line with a previous study in Uganda [33]. The data regarding any relationship between infant gender and early initiation of breastfeeding are contradictory, previous studies in Turkey and India report that boys were fed earlier than girls [34, 35]. This could be explained by the difference in cultures, for example, in Sudan, it was observed after delivery of a male baby, the family members would give him more care in comparison to a female baby by handling the male baby first and then pass him to the mother for breastfeeding and this may cause delaying of initiation of breastfeeding among male babies. Moreover, male infants are at higher risk of preterm birth and morbidity, respectively [36, 37] and these factors are associated with delayed initiation of breastfeeding [38–40]. Although, the gestational age at birth was not mentioned, in one hospital in Sudan, high rates of preterm births were reported in 2010 and about one fifth of the total preterm births identified as medically indicated (iatrogenic) [41]. This could be explained by maternal medical disorders such as preeclampsia and premature infants requiring hospitalization, both circumstances which may lead to delayed initiation of breastfeeding.

The study found that mothers with medical disorders and their infants were more likely to experience delayed initiation of breastfeeding. Inconsistent with the current results, delayed initiation of breastfeeding was observed among mothers with medical disorders such as diabetes and hypertension [18, 42]. In an African context, among maternal medical disorders, maternal human immunodeficiency virus (HIV) was found as a key barrier to early initiation of breastfeeding [43–45]. In Mauritius, despite the fact that 60.6% of mothers initiate breastfeeding, low rate of exclusive breastfeeding in the first six months were documented (17.9%) [46]. Several studies identify the positive influence of breastfeeding education for successful breastfeeding [47–49].

In spite of a high rate (17.2%) of caesarean delivery in this study, an association between caesarean delivery and early initiation of breastfeeding was not identified. This may be because the sample size was not powered to show a difference. Previous studies have shown that caesarean delivery was a key barrier for early initiation of breastfeeding in different settings [18, 21, 22, 33, 43, 50–52]. When compared to planned caesarean delivery, emergency caesarean delivery was found to be associated with delayed initiation of breastfeeding [53]. Further categorizations of caesarean delivery (planned/ emergency) are vital to be taken into consideration in future research [53].

This study show no association between the delayed initiation of breastfeeding and child hospitalization. This might be explained by the poor outcomes for the hospitalised children (i.e. the deaths) which the current study failed to trace. A larger sample may have shown evidence for such an association. For future research in this area larger sample size, more detail about the main reasons for hospitalization and inclusion of both mothers in the community in addition to those in hospital would be of value.

Although we have found that 59% of the mothers received breastfeeding education during the pregnancy course and/or after the delivery in logistic regression breastfeeding education was not found to be associated with breastfeeding initiation. Previous studies have shown that mothers who have received breastfeeding education were more likely to have initiate early breastfeeding [54, 55]. This could be explained by the fact that, even among the mothers who had received information about breastfeeding by healthcare personnel, more than half of them learned about breastfeeding from family members as well. Perhaps the influence of the family member on the mothers was more powerful than healthcare personnel. For example, in Myanmar, traditional beliefs that exclusive breastfeeding is not sufficient for babies and that solid foods and water were necessary are identified as barriers to the initiation of breastfeeding [56]. Moreover, in Sudan, even among healthcare workers, capacity-building regarding breastfeeding practices need to be improved [57]. To ensure the reliability of breastfeeding information, healthcare workers may require education about the provision of breastfeeding education. Involving family members in discussion about breastfeeding in a respectful and inclusive manner may help correct inaccurate information and help to share accurate information in a culturally appropriate manner.

In the current study, there was no association between maternal age, parental educational level or occupation and the initiation of breastfeeding. Previous studies have shown that parental education [54, 55, 58–60], occupation [59] and maternal age [58] were associated with initiation of breastfeeding. The variations of the associated factors among studies could be due to the influence of such factors on the family socioeconomic status. For example, paternal occupation could influence early initiation of breastfeeding through improving family income as high socioeconomic status was found to be associated with early initiation of breastfeeding [35, 61–64].

Although the sample size of the employed mothers among the study participants was small in comparison to studies in Ghana and Uganda [22, 65], this study highlighted two main issues, the low employment rate among the study participants and the lack of support for breastfeeding mothers who are employed. For example, some mothers faced breastfeeding problems related to work, these could be explained by several factors including poorly paid work, limited breastfeeding support in the workplace, excessive travel distance requiring long journeys between home and work and limited access to maternity leave. Further research with a larger sample size of the mothers of infants and young children would allow further exploration of the challenges experienced by women who wish to breastfeed and participate in the paid workforce.

### Limitations

Study limitations included recall bias as mothers of children aged up to two years were asked for early infant feeding detail. Not tracing infant maturity status (term or preterm), gestational age, birthweight, reasons for and outcomes of infant hospitalization limited study analysis and outcomes - particularly as a recent systematic review and meta-analysis documented poor infant survival associated with delayed breastfeeding initiation [66].

## Conclusion

The study concluded that the prevalence of early initiation of breastfeeding was 87.2% among mothers with children of two years or less in Kassala, Eastern Sudan. However, an association with delayed initiation of breastfeeding was found amongst mothers who had medical disorders and those who had a male infant. Wherever possible, early initiation of breastfeeding should be promoted for all infants’ regardless of gender.

## Abbreviations

AORs: Adjusted Odds Ratios
CI: confidence interval
SD: standard deviation
WHO: World Health Organization
